# The Interplay of Dietary Fibers and Intestinal Microbiota Affects Type 2 Diabetes by Generating Short-Chain Fatty Acids

**DOI:** 10.3390/foods12051023

**Published:** 2023-02-28

**Authors:** Muhammad Mazhar, Yong Zhu, Likang Qin

**Affiliations:** 1 Key Laboratory of Plant Resource Conservation and Germplasm Innovation in Mountainous Region (Ministry of Education), College of Life Sciences/Institute of Agro-Bioengineering, Guizhou University, Guiyang 550025, China; 2 School of Liquor and Food Engineering, Guizhou University, Guiyang 550025, China

**Keywords:** dietary fibers, intestinal microbiota, short-chain fatty acids, fermentation, type 2 diabetes

## Abstract

Foods contain dietary fibers which can be classified into soluble and insoluble forms. The nutritional composition of fast foods is considered unhealthy because it negatively affects the production of short-chain fatty acids (SCFAs). Dietary fiber is resistant to digestive enzymes in the gut, which modulates the anaerobic intestinal microbiota (AIM) and fabricates SCFAs. Acetate, butyrate, and propionate are dominant in the gut and are generated via Wood–Ljungdahl and acrylate pathways. In pancreatic dysfunction, the release of insulin/glucagon is impaired, leading to hyperglycemia. SCFAs enhance insulin sensitivity or secretion, beta-cell function, leptin release, mitochondrial function, and intestinal gluconeogenesis in human organs, which positively affects type 2 diabetes (T2D). Research models have shown that SCFAs either enhance the release of peptide YY (PYY) and glucagon-like peptide-1 (GLP-1) from L-cells (entero-endocrine), or promotes the release of leptin hormone in adipose tissues through G-protein receptors GPR-41 and GPR-43. Dietary fiber is a component that influences the production of SCFAs by AIM, which may have beneficial effects on T2D. This review focuses on the effectiveness of dietary fiber in producing SCFAs in the colon by the AIM as well as the health-promoting effects on T2D.

## 1. Introduction

The gut microbiota (GM) is a complicated and dynamic ecosystem that interacts with the host, maintaining a mutualistic relationship. The microbes can influence numerous physiological mechanisms, including those involved in glucose regulation, lipid metabolism, pathogen resistance, and micronutrient production [1]. Thus, modulating GM might be a reasonable approach to preventing inflammatory and metabolic diseases. For example, previous studies employing animal models showed that modulating GM had salutary effects on obesity, insulin sensitivity, and type 2 diabetes (T2D) [2,3]. However, no evidence was found that specific microbial communities were directly linked to these diseases, while some evidence suggested that gut microbial activity is beneficially associated with T2D [4]. In addition, the composition of GM is influenced by internal and external factors. Genetics plays a major role in elucidating the gut microbial composition, and several potential strategies have been employed to induce positive changes in gut microbial communities via fermentative activity [5]. The GM is an essential storehouse for human health; 60 bacterial phyla have been identified in the human gastrointestinal tract (GIT) (including Firmicutes, Bacteroides, Actinobacteria, Fusobacteria, Proteobacteria, Verrucomicrobia, Cyanobacteria, and Spirochaetes) [6].

**Figure 1:**
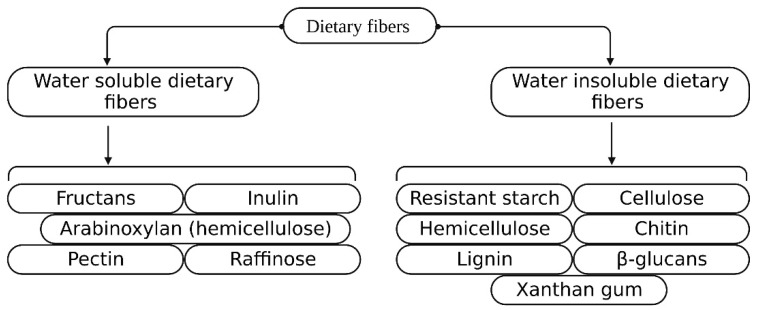
Dietary fiber classification based on water solubility/insolubility.

Dietary fiber is an essential component of food composed of a complex polymer of phenylpropanoid units [7,8]. It is mainly classified into four subgroups: resistant starches, lignins, resistant oligosaccharides (galacto-oligosaccharides, fructo-oligosaccharides, etc.), and non-starchy polysaccharides (cellulose, hemicellulose, and pectin) [9]. Soluble fiber is resistant to gastrointestinal digestive enzymes and is utilized by the anaerobic intestinal microbiota (AIM) to produce short-chain fatty acids (SCFAs), whereas insoluble fiber is not degraded or utilized by the human GIT [9,10]. Regular consumption of soluble dietary fiber may modulate the intestinal microbiota, positively affecting T2D [11,12,13,14]. Legumes are considered to have an excess amount of fibers including resistant starch (RS) [15,16]. The recommended amount of dietary fiber by the World Health Organization, Food and Agriculture Organization (WHO/FAO), and European Food Safety Authority (EFSA) is 25 g/day, depending on the laxation of healthy individuals [17,18]. Intervention with dietary fiber in the AIM has numerous benefits for human health, such as energy consumption, AIM integrity, and regulation of immune functions; arabinoxylan has shown beneficial effects in T2D, linked to AIM amendment and metabolites produced during fermentation [19]. Types of water-soluble/insoluble dietary fiber are shown in Figure 1.

SCFAs are a sub-class of fatty acids composed of carbon atoms (six or fewer), acetate (C2), propionate (C3), butyrate (C4), pentanoic acid (C5), and hexanoic acid (C6) [20]. Among them, the focus is primarily on acetate, propionate, and butyrate due to their excess production in GIT [21]. These fatty acids are generated from dietary fiber through fermentation via the AIM in the mammalian colon and have shown beneficial effects on metabolic activity [22]. The consumption of excess amounts of starchy foods and avoidance of physical exercise leads to the disruption of energy balance and generates intricate symptoms, collectively called metabolic syndrome; hypertension, obesity, glycemic imbalance, and T2D are typical manifestations of metabolic syndrome [23]. 

The higher intake of dietary fibers affects T2D, non-digestible oligosaccharides are fermented by gut microbial communities, producing SCFAs, which may positively contribute to different organs of the body [24]. Several systematic reviews have previously focused on fibers, lifestyle interventions, probiotics, and fecal microbial transplantation, and its effects on T2D [25,26]. This review focuses on the effectiveness of fiber in producing SCFAs in the colon by the AIM as well as the health-promoting effects in T2D, and the negative effects of fast-food consumption on T2D have also been addressed. This review provides a reference for subsequent research. 

## 2. Fibers

### 2.1. Dietary Fiber

Dietary fiber, comprising endogenous non-digestible carbohydrates and lignins, is an essential component of plants [23]. The types of fiber differ in their anaerobic fermentability, viscosity, chemical structure, and solubility in water [27]. Such fiber consists of the polymers of carbohydrates, combining monomeric units (three or more), which are not hydrolyzed/absorbed in the human gut when exposed to digestive enzymes [1]. Dietary fiber is classified into two categories, soluble and insoluble. Soluble dietary fiber is preferable because it is metabolized by the AIM, producing SCFAs [28]. Firmicutes and Actinobacteria species are considered to especially respond to dietary fiber [1,29]. 

The endogenous components of plant-based foods have been well studied for the last few decades, in which dietary fibers positively affect the human host. Meta-analysis showed that the consumption of galacto-oligosaccharides and fructans may enhance the biodiversity of *Lactobacillus* and *Bifidobacterium* species in the gut [30]. Dietary fiber directly influences the production of SCFAs in the human gut (lumen) [11,31]. An increase in dietary fiber increases the production of SCFAs and vice versa. Low intake of dietary fiber can also affect the production of amino acids and mucins, which reduces energy production for metabolic activity [1,29]. Previous studies have shown that high fiber intake leads to higher production of SCFAs (acetate, propionate, and butyrate) and vice versa [31]. In a pilot study on obese volunteers investigating rice bran/cooked navy beans (rich in dietary fiber), the results showed that the number of SCFAs (acetate and butyrate) was increased while the Firmicutes to Bacteroidetes proportion was decreased [31,32]. Previous studies investigated whether dietary fiber from whole grains and/or vegetables/fruits affects inflammatory markers and the composition of GM. Whole grain showed a significant decrease in lipopolysaccharide (LPS) and tumor necrosis factor α (TNF-α), while vegetables/fruits showed substantial changes in interleukin-6 (IL-6) [33,34].

### 2.2. Prebiotic Inulin

Inulin belongs to a class of dietary fiber called fructans, which are produced in plants [35,36]. Being prebiotic, the components of fructans are effectively modulated in the AIM (with *Bifidobacterium* spp. dominating) and positively respond in T2D patients via the production of SCFAs (acetic and propionic acids) in the ileum of the GIT [37]. Inulin-type fructans (ITFs) are systemically beneficial, promoting AIM growth and producing H_2_S, CO_2_, and organic acids. Fructans also have beneficial effects on metabolic syndrome, including T2D [38]. ITFs modulate GM and increase the production of SCFAs (acetic and propionic acids) [38], which improves the level of hemoglobin Alc by accelerating glucagon-like peptide-1 (GLP-1) production, resulting in the reduction of harmful compounds such as H_2_S and indole [39]. Moreover, ITFs may also regulate inflammation associated with LPS, IL-6, TNF-α, and interferon-γ [40,41].

### 2.3. Resistant Starch

Starches are complex polysaccharides in the form of grains stored in roots, seeds, and fruits [42,43]. These polysaccharides are present in the human diet in maize, cassava, potatoes, rice, and wheat [44,45]. Regarding digestibility, starches are classified into three classes: resistant starch (RS), slowly digestible starch (SDS), and rapidly digestible starch (RDS) [46,47]. RS is the fraction of starch that is indigestible by gut digestive enzymes, fermented by the AIM, and known to produce SCFAs [48,49,50]. The term “resistant starch” was first used by Englyst in the 1980s [51], and the efficiency of resistant starch with regard to prebiotics and lipid/glucose metabolism was studied concerning the gut environment [52,53,54,55,56,57,58]. As a component of functional foods, an indigestible portion of RS is categorized as dietary fiber. RS-1, RS-2, RS-3, RS-4, and RS-5 are sub-types of RS, among which RS-1 is substantially inaccessible, i.e., it has intact cell walls (encapsulated) that prevent access by digestive enzymes [59]. RS-2 comprises starch granules with crystalline polymers (B- or C-); this type of starch lacks water channels, and due to the condensed surface, it provides fewer sites for digestive enzymes [52]. RS-3 contains retrograded starch, which is normally found in cooked food (plant-based), and the retrograde/double helix structure of RS-3 starch molecules prevents attachment to digestive enzymes [60]. The functional group RS-4 acts by restraining the attachment of digestive enzymes [61]. The configuration of amylose-lipid complexes in RS-5 prevents it from fitting into the binding pockets of digestive enzymes [62]. Foods containing high RS content are significantly beneficial for human health because the fermentation of RS in the colon produces SCFAs by AIM, and the quantity of acetic, butyric, and propionic acids is higher than the quantity of iso-butyric, valerian, and iso-valeric acids [63]. Previous studies showed numerous effects of SCFAs on human health, including reduced cholesterol and triglyceride levels in blood and providing a significant amount of energy to colonocytes, which balance the status of the colonic epithelial lining [64,65,66,67]. In addition, SCFAs are beneficial in terms of glucose reduction and insulin secretion, and show positive effects on T2D [64,68,69,70,71]. 

## 3. Dietary Fiber, Inflammatory Markers, and T2D 

T2D is among the major diseases associated with a low level of inflammatory processes, characterized by amendments in the secretion of cytokines [72]. The amount of inflammatory markers (IL-6, TNF-α, and LPS) in T2D is increased, which is associated with dysfunction in insulin resistance and β-cell activity, and the amount of LPS in diabetic patients is twice as high as that in healthy individuals [73]. A high-fat diet is associated with metabolic endotoxemia caused by serum LPS, resulting in obesity and insulin resistance [74], and high serum LPS enhances TNF-α and inhibits insulin signals [75]. An excess amount of ANK-α indirectly inhibits insulin signaling by serine-307 phosphorylation in the substrate of the insulin receptor [76]. According to scientific reports, the composition of the diet can positively affect the inflammatory process; *Lactobacillus* spp. and *Bifidobacterium* spp., which are stimulated by dietary fiber, show anti-inflammatory properties [77]. Dietary fiber at 40 g/day can reduce the level of TNF-α [78]. 

## 4. Effects of Fructose on SCFAs and T2D

Sugar is an important source of energy in our daily diet, and there is increasing evidence that high sugar intake causes a number of major diet-related health problems, such as T2D and obesity [79,80]. Dietary factors influence blood glucose homeostasis in T2D; blood glucose levels rise when fructose is converted into glucose in the liver. This conversion takes time, so a small portion of fructose is converted into glucose, resulting in a lower increase in blood glucose levels [81]; therefore, the glycemic index of fructose is only 23 [82]. In addition to contributing to blood glucose homeostasis, fructose has also been shown to improve glycemic control at moderate levels [83,84]. The health effects of fructose are closely related to the consumption amount. Ultimately, it was determined that a high-fructose diet and a certain gut microbiota profile may be associated with the inflammation of the liver, pancreas, and colon. With low or inadequate fructose intake, no adverse effects were found on body weight, fasting blood glucose, histology, gut microbiota, or colonic SCFA levels [85,86,87]. Some evidence showed that fructose causes insulin resistance in the liver, which can negatively impact blood glucose homeostasis [88]. 

## 5. Effects of Lipids on SCFAs and T2D

A lipid molecule is mostly made up of repeating units named fatty acids. There are two types of fatty acids, saturated and unsaturated. Humans get most of their energy from fatty acids, which are the main components of triacylglycerols found in oils and fats [89,90]. Long-term consumption of a high-fat diet affects gut microbiota composition in animal models as well as in humans, which directly impacts SCFA production and host health [91]. High-fat diets containing medium-chain fatty acids, monounsaturated fatty acids, and polyunsaturated fatty acids, low-fat diets containing long-chain fatty acids, and diets with high Bacteroidetes or Firmicutes ratios were associated with increased SCFA production [92].

## 6. Short-Chain Fatty Acids (SCFAs)

SCFAs are organic acids produced in the human gut, where the AIM resides [70]. Quantitatively, these fatty acids are measured in millimoles, and they are predominately represented by acetate, butyrate, and propionate [93]. These three SCFAs are discussed in the current review. The dietary carbon flow is based on SCFAs [94], and their production is fairly well understood and characterized [95,96]. The ratio and concentration of SCFAs depend on the microbial composition and the substrate (dietary fiber) provided to the GM [97]. The molar ratio of acetate, propionate, and butyrate is 3:1:1. SCFAs constitute 90–95% of the colon, whereas formic acid is present in a smaller proportion [93]. As a result of antibiotic treatment depleting the microbiota, mice were found to produce lower amounts of SCFAs, compared to mice that did not receive antibiotics [98]. A diet rich in prebiotics may be particularly effective at increasing SCFA production in diabetes [99]. According to previous studies, individuals with T2D have lower proportions of microbiota species producing butyrate [99,100]. Some of the beneficial properties of SCFAs that positively affect human health are shown in Table 1.

### 6.1. The Contribution of Gut Microbiota Producing SCFAs

Dietary fibers are resistant to gut digestive enzymes, which contribute to the production of SCFAs during colonic fermentation [104]. Acetate, propionate, and butyrate are dominant SCFAs in the gut [77]. These fatty acids are composed of 1–6 carbon atoms and are naturally saturated [29]. Present-day research has shown a significant role for AIM, and the metabolites produced during dietary fiber fermentation positively contribute to T2D [105]. Gut intestinal microbiota, including *Clostridiales* spp. SS3/4, *Roseburia inulinivorans*, *Roseburia intestinalis*, *Faecalibacterium prausnitzii*, and *Eubacterium rectale*, produce butyrate, which has a protective role in T2D, even though these species are decreased in diabetes [106]. In addition, oral administration of *Clostridium butyricum* in obese diabetic rats was found to modulate gut microbiota to produce butyrate, leading to reduced proportions of Bacteroides and Firmicutes spp. [107]. 

In diet-induced diabetes, chitosan and antibiotics targeting Gram-negative intestinal microbes may be considered antidiabetic agents [108,109]. Cross-feeding GM metabolizes lactate into acetate, propionate, and butyrate in the gut fermentation process, in which propionate and butyrate are produced in limited quantities owing to selected GM, while acetate is a regular product in the gut [94]. Propionate is produced during the fermentation of propiogenic substrate (fucose/rhamnose) by *Akkermansia municiphilla*, whereas butyrate is produced through RS fermentation by *Eubacterium hallii*, *Eubacterium rectale*, *Faecalibacterium prausnitzii*, and *Ruminococcus bromii* in the gut; moreover, butyrogenic bacteria ferment pyruvate, lactate, and acetate into butyrate [93]. Acetate, propionate, and butyrate are the energy sources for the human body. Butyrate is directly utilized in the liver, heart, brain, and colon; propionate is used for gluconeogenesis in the liver, and acetate is used as fuel in peripheral tissues [110]. 

The responsiveness of free fatty acid receptors (FFAR-2 and -3) is proportional to the length of the carbon chain. For example, acetate and propionate are more responsive to FFAR-2, whereas butyrate and propionate are more responsive to FFAR-3 [111]. Medium (FFAR-1) and long-chain (FFAR-4) fatty acids were found to positively respond to inflammation and insulin secretion [112]. FFAR-1 enhances specific pancreatic β-cell activity, while in T2D, this activity is downregulated, resulting in FFAR-1 inhibition and insulin resistance [113]. FFAR-4 boosts these fatty acids (unsaturated) to stimulate glucagon-like peptide-1 (GLP-1), secreting insulin from β-cells [114]. Propionate and butyrate may positively regulate obesity and T2D when administered orally [115,116,117].

### 6.2. Production of SCFAs via Anaerobic Bacterial Pathways and the Role of Akkermansia Muciniphila in T2D

**Figure 2:**
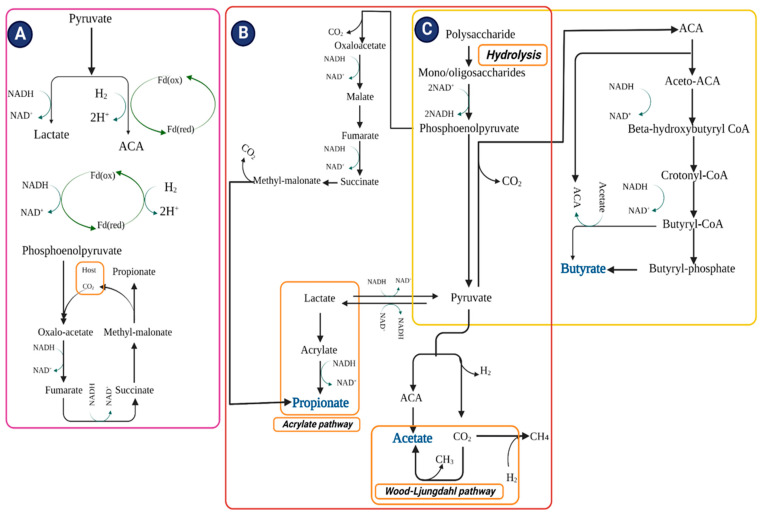
Schematic presentation of three pathways that contribute to disposing of excess reducing equivalents. (**A**) Reduction of pyruvate into lactate, thereby reducing NADH, pyruvate; ferredoxin oxidoreductase and hydrogenase/NADH; ferredoxin oxidoreductase and hydrogenase dispose of reducing equivalents into molecular hydrogen and NADH is reduced via electron transport chain. (**B**) Acetate is formed directly from acetyl CoA through the Wood–Ljungdahl pathway. Propionate is formed from PEP via succinate decarboxylation pathway or acrylate pathway while reducing propionate. (**C**) Butyrate is shaped by condensing acetyl CoA (two molecules) in the presence of butyrate kinase or by employing exogenously derived acetate through butyryl-CoA: acetate-CoA transferase. Abbreviations: NADH; nicotinamide adenine dinucleotide (NAD) + hydrogen (H): ACA; acetyl coenzyme A: H; Hydrogen: CO_2_; carbon dioxide: CH_3_; methyl radical: CH_4_; methane.

The non-digestible carbohydrates are hydrolyzed by the AIM into monosaccharides and oligosaccharides during anaerobic fermentation in the colon [118]. For the metabolization of monosaccharides into phosphoenolpyruvate (PEP), the Embden–Meyerhof–Parnas pathway (sugars containing 6-c) and the pentose phosphate pathway (sugars containing 5-c) are utilized [95]. Eventually, organic acids/alcohols are formed during PEP fermentation. Nicotinamide adenine dinucleotide (NAD) + hydrogen (H) (NADH) is produced during the reaction of an acidic protein, glyceraldehyde-3-phosphate dehydrogenase (GAPDH). Three pathways contribute to the disposal of excess reducing equivalents, as presented in Figure 2A. First is the traditional fermentation pathway, in which lactate/ethanol is produced from the reduction of pyruvate. Second, pyruvate is reduced to acetyl-CoA (ACA) and NADH to NAD^+^ [119]. The second pathway produces excess amounts of H_2_ molecules by using two major routes, pyruvate (exergonic) and NADH (endergonic) via ferredoxin oxidoreductase and hydrogenase, respectively. Despite depleting/consuming H_2_ molecules, the AIM is a primary participant in the fermentation process when H_2_ pressure in the large intestine (lumen) is low [120]. Third, the fundamental electron transport chain (ETC) proceeds with anaerobes, starting with PEP carboxylation and the reduction of oxaloacetate into fumarate [121]. The electrons are accepted by fumarate from NADH; NADH dehydrogenase and fumarate reductase constituted an ordinary electron transfer chain (OETC) [121,122]. NADH-dehydrogenase contributes to the transport of protons across the cell membrane, resulting in the chemiosmotic synthesis of ATP. Succinate (produced by fumarate reductase) is transformed into methylmalonate once the preferential load of CO_2_ is reduced. PEP can also be recycled from oxaloacetate through the carboxylation process. 

SCFAs are the end product of the fermentation pathways. Pyruvate is transformed into ACA, releasing H_2_ and CO_2_ molecules. Hydrolysis of ACA leads to the formation of acetate, or it can also be produced by the Wood–Ljungdahl pathway utilizing CO_2_, wherein CO_2_ is reduced to CO coupled with CoASH and a methyl group and converted to ACA [123,124]. Propionate is formed either by utilizing PEP via OETC or by reducing lactate to propionate via the acrylate pathway [95]. These pathways accommodate supplementary NADH associated with lactate fermentation (Figure 2B). The condensation of ACA (2 molecules) results in the formation of butyrate, which is subsequently reduced to butyryl CoA (Figure 2C). ACA is produced from lactate, and then lactate is utilized by gut bacteria to produce butyrate [125]. Two pathways are involved in the formation of butyrate: the traditional pathway uses phosphotransbutyrylase and butyrate kinase to convert butyryl CoA into butyrate, accompanying ATP formation, and in the alternative pathway, butyryl CoA is converted to butyrate via butyryl-CoA: acetate CoA transferase [126,127]. The exogenic utilization of acetate to form butyrate and ACA involves cross-feeding among acetate and butyrate-producing bacteria [128,129]; the human GM prefers the alternative over the traditional pathway [126]. 

The symbiotic association between GM and the human body is significant in SCFA production [130]. The primary metabolites (H_2_ molecules) produced to get acetate must be utilized by secondary fermenters to reduce the burden of these molecules and accelerate the oxidation of NADH via primary fermenters [131]. The human body provides the CO_2_ molecules required in the OETC, and an average of 0.7 kg/day of CO_2_ is produced by the human organism [132]. By exchanging SCFA anions, some of that production is secreted into the gut (lumen) as HCO_3_, which is likely a significant pH-regulating mechanism, since protons in the gut (lumen) generated during the formation of SCFAs are neutralized by bicarbonate to produce CO_2_ [131]. Subsequently, much is known about the biochemistry of SCFA production from carbohydrates via the AIM. However, further study is still needed to determine whether SCFAs, as the significant output of indigestible carbohydrates via the AIM, have beneficial effects in T2D.

*Akkermansia muciniphila* is the only representative Gram-negative *Verrucomicrobia* inhabiting human intestinal mucosa [133]. In the studies by Derrien, gene sequence analysis revealed that multiple genes are associated with mucin encoding, and a single chromosome containing 2176 genes with 55.8% GC content was found in the MucT type strain of *A. muciniphila* (ATCC BAA-835 1/4 CIP107961T) [134,135]. This immobile, oval-shaped microorganism is purely anaerobic and contains chemical organotrophic material that can endure low levels of oxygen. The enzymes produced by *A. muciniphila* were responsible for the breakdown of mucin, and the mucin in the mucosal layer of the epithelium was used as a source of carbon and nitrogen. In order to release the sulfate, *A. muciniphila* splits these compounds into acetic and propionic compounds [136,137]. According to an analysis of its 16SrRNA signature, *A. muciniphila* makes up 3 to 5% of the gut microbiome even in healthy adults, but the amount depends on several factors. Age has been closely associated with stability in humans. This species begins to colonize at a young age and ranges from 5.0 to 8.8 log cells/g in a year, which is comparable to the adult stage, although it decreases with age [138,139]. The combined effects of an excess amount of *A. muciniphila* supplementation can positively affect metabolic disorders including T2D, and early vancomycin therapy may help control the progression of autoimmune diabetes by early colonization of the intestinal tract with *A. muciniphila* [140,141]. 

### 6.3. Effects of SCFAs on T2D 

**Figure 3:**
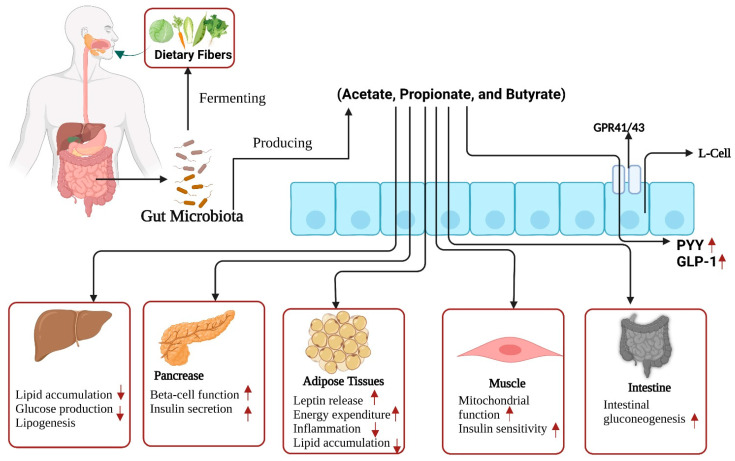
Gut microbiota ferments dietary fiber and produces short-chain fatty acids (SCFAs) acetate, propionate, and butyrate. These SCFAs may facilitate the production/release of GLP-1 and PYY from enteroendocrine (L-cells) and activate adipose tissue to release leptin hormone. These fatty acids also enhance insulin sensitivity and mitochondrial functions in muscle cells, promote pancreatic functions, including insulin secretion and beta cell activity, and promote intestinal gluconeogenesis. In the liver, lipid accumulation, and glucose production are reduced. Arrows pointing upwards indicate an increase, and arrows pointing downwards indicate a decrease. Abbreviations: PYY; peptide YY: GLP-1; glucagon-like peptide-1: GPR-41; G-protein receptors-41: GPR-43; G-protein receptors-43.

SCFAs are metabolites of gut microbe fermentation that result from indigestible dietary fiber and may have a beneficial role in T2D [142]. Compared to normal animals, diabetic rodents that consumed a high-fat diet with streptozotocin showed lower levels of acetate, propionate, and butyrate [143,144]. It was found that T2D patients had lower fecal butyrate and propionate concentrations, as well as acetate concentrations, than healthy subjects [145]. Improved insulin secretion/sensitivity, reduced fat accumulation, intestinal gluconeogenesis (IGN) triggering, and inflammation are the mechanisms by which SCFAs can positively affect T2D (Figure 3) [70,146]. A study using homeostatic model assessment of insulin resistance (HOMA-IR) observed an adverse correlation between blood insulin levels and total SCFAs, including acetate and propionate [147]. In vitro and in vivo studies showed that propionate can enhance the release of glucose-stimulated insulin, sustain β-cell mass by decreasing trans-differentiation in α-cells, obstruct apoptosis, and assist in proliferation [70,148]. Moreover, it was shown in mouse models that butyrate improved insulin sensitivity [116,149]. These mechanisms support energy consumption and boost mitochondrial functions [116]. 

Propionate- or butyrate-induced IGN affects glucose homeostasis, the cAMP-dependent pathway, and the gut–brain neural circuit [150]. Acetate enhances the suppression of lipogenesis in the liver and decreases lipid aggregation in adipose tissues, while glucose transporter-4 genes and myoglobin are enhanced in the abdominal muscles of diabetic rats [23]. The peroxisome proliferator-activated receptor-α (PPAR-α) gene was upregulated in the presence of acetate, which may suppress body fat aggregation [151,152,153]. Furthermore, SCFA supplementation reduces hepatic steatosis and body weight [154]. In vitro and in vivo models showed that SCFAs either enhance the release of peptide YY (PYY) and GLP-1 from L-cells (entero-endocrine), or promote the release of leptin hormone satiation in adipose tissues through G-protein receptors (GPR-41 and/or GPR-43) [155,156,157,158]. SCFAs promote lipid oxidation and energy consumption and were found to increase fasting fat oxidation and PYY concentration during colonic infusion in obese subjects [159]. Butyrate may weaken inflammation generated by the interaction of macrophages and adipocytes by decreasing lipolysis and obstructing inflammatory signals [160]. These fatty acids showed beneficial effects on T2D by reducing the production of TNK-α, IL-6, and monocyte chemoattractant protein-1 (MCP-1); nuclear factor kappa-B (NF-κB) activity was also constrained. Propionate had a positive influence on T2D, participating in the downregulation of inflammatory chemokines and cytokines, such as CC chemokine ligand-5 (CCL-5) and TNF-α [161].

## 7. Fast Foods

Fast foods are a commercial term used to describe foods sold in restaurants and stores that contain frozen, pre-cooked, or pre-heated ingredients and are sold as takeout [162]. Fast-food consumption is associated with higher energy, fat, sodium, and sugar intake, along with a lower intake of fruits, vegetables, and fibers [163]. Fast foods also tend to have higher energy density and lower nutritional quality compared to home-cooked meals and recommended diets [164,165]. China is one of the most populous countries, and the consumption of fast food is increasing day by day. As a result of the rapid growth of the fast-food industry and fast-food consumption in China, public health concerns have arisen about adverse health effects, such as obesity [166,167].

### 7.1. Effects of Fast Foods on Gut Microbiota and SCFAs 

Fast foods contain low dietary fiber and high fats, which negatively influence the gut microbiota. The composition of gut microbiota is also affected by the quantity and quality of dietary fats [168]. In addition, fast foods are one of the main sources of toxic heavy metals in humans, especially children [169]. The non-essential metals chromium (Cr), cadmium (Cd), nickel (Ni), and lead (Pb) are toxic when they bioaccumulate in tissues and cause inflammation and other effects [170]. In mice that were fed high-fat, low-fiber diets, *Bacteroides* were less likely to develop and *Firmicutes* and *Proteobacteria* were more likely to develop [171]. The gut microbiota plays a very important role in food absorption and mild inflammation, contributing to the development of obesity and diabetes mellitus. Several metabolic pathways are influenced by gut microbiota metabolites (SCFAs), including insulin signaling, incretin production, and inflammation [172,173,174]. In general, fast-food products tend to contain large amounts of manufactured trans-fatty acids, and in people with diabetes, particularly those who eat a diet with high trans-fatty acids, more pro-inflammatory molecules are produced [175,176,177]. 

Cooking or heat treatment can significantly change the composition and structure of food; in fact, the physicochemical properties of food can be altered by heat, which can degrade antimicrobial compounds [178]. The heating process leads to the production of new compounds, some of which has prebiotic properties and affects the composition of gut bacteria. For instance, a relative decrease in bacterial groups such as *Lactobacillus*, *Bifidobacterium*, *Akkermansia*, *Parasutterella*, *Barnesiella Dorea*, *Oscillibacter*, and *Alistipes* was observed in animals fed with melanoidin-enriched malt [174,179]. Thus, the amount of fiber is affected by the consumption of fast food, and the number of gut microbes decreases, leading to a decrease in SCFAs. 

### 7.2. Effect of Fast Foods on Diabetes 

The term “junk food” refers to a variety of processed foods, fast foods, and ready-made snacks. Fast foods, which are heavily processed, have an adverse effect on health. Eating fast food and eating out are major risk factors in terms of poor diet quality, higher calories, fat intake, and lower dietary micronutrient density [180]. Currently, there are no government policies controlling fast-food pricing or advertising in some countries, leading to the opening of new global chains. Eating fast food twice a week has been shown to increase the risk of insulin resistance and T2D [181]. Obesity, abdominal fat gain, lipid and lipoprotein disorders, impaired insulin, glucose homeostasis, systemic inflammation, as well as oxidative stress, have been associated with frequent fast-food consumption [182]. A positive correlation between fast-food restaurants and the prevalence of diabetes was found in all counties except those with high poverty or middle minority populations [183]. A previous study showed that consuming excess calories shortens the lifespan, while moderate calorie restriction slows the aging process and protects the body and brain from age-related damage [184]. T2D is strongly associated with overweight and obesity. Animal studies have shown that nitrosamines in fast food are toxic to beta cells and increase the risk of T2D [185]. A previous study found that primiparous women who ate more fast food before pregnancy had an increased risk of developing diabetes during pregnancy and giving birth to a child with low birth weight [186]. Several chronic metabolic disorders may develop due to the consumption of fast foods, including hyperglycemia, glycosuria, hyperlipemia, negative nitrogen balance, and sometimes ketonemia, and junk food consumption causes over 90% of T2D cases [187]. 

## 8. Conclusions and Future Perspectives

Promoting health and preventing disease rely on maintaining a balance between the intestinal microbiome, genetic factors, environmental factors, and dietary conditions that affect substrate availability. Intestinal bacteria produce SCFAs as metabolites, and their concentration depends on the composition and the population size of these microorganisms. In addition to their effects on digestion, many studies have investigated how SCFAs produced by intestinal microbiomes affect organs and tissues elsewhere in the body. In addition, regular consumption of fast food negatively affects the production of SCFAs, and GM survival is responsible for SCFA production. Dietary fiber, as an essential component of foods, can be classified into soluble and insoluble forms. Soluble dietary fiber is resistant to gut-digesting enzymes and fermented by the AIM, resulting in the production of SCFAs (acetate, propionate, and butyrate). 

In this review, the effectiveness of dietary fiber in producing SCFAs in the colon by the AIM as well as its health-promoting effects on T2D patients were discussed. Several pathways lead to the production of acetate, propionate, and butyrate, including the Wood–Ljungdahl pathway, the succinate decarboxylation pathway, and the acrylate pathway. Both in vitro and in vivo studies have demonstrated that SCFAs increase the release of PYY and GLP-1 from L-cells and promote leptin hormone satiation in adipose tissue via G-protein receptors such as GPR (41 and 43). SCFAs have been shown to have beneficial effects on T2D by reducing the production of TNK-α, IL-6, MCP-1, and NF-κB; and propionate has a positive impact on T2D through the downregulation of inflammatory chemokines and cytokines such as CCL-5 and TNF-α. These combined effects lead to a positive influence on T2D. In the future, the effectiveness of GM intervention in T2D will be verified by clinical trials, and the advantages will be explored. There is an urgent need for research in this area for human populations.

## Figures and Tables

**Table 1:** Important health benefits of short-chain fatty acids in modulating gut microbiota.

SCFA	Chemical Formula	Molar Mass (g/mol)	Precursor	Producers	Effects on Human Health	References
Acetate	CH_3_COOH	60.05	Pyruvate	*Streptococcus*, *Bifidobacterium*, *prevotella*, *species*, *Blautia hydrogentrophica*, *and Akkermansia muciniphilia*	Inhibits *Escherichia coli* O157:H7 infections Participates in cholesterol synthesis	[29]
Propionate	CH_3_CH_2_COOH	74.08	Phosphoenol pyruvate	*Akkermansia muciniphilia*, *Eubacterium halli*, *Phascolarctobacterium succcinatutens and Clostridium*, *Ruminococcus species*	Reduces cholesterol in the liver Enhances lipid metabolism	[29,101]
Butyrate	CH_3_(CH_2_)_2_COOH	88.11	Deoxyhexose ACA	*Roseburia intestinalis*, *Faecalibacterium prausnitizii*, *Eubacterium rectale*, *Coprococcus eutactus*, *and Clostridium symbiosum*	Enhances MUC2-gene expression and produces an excess amount of mucin Acts as a source of energy (70%) for intestinal epithelial cells Is efficient against tumor cells and boosts apoptosis	[29,101,102,103]

## Data Availability

The original contributions presented in this study are included in the article/supplementary material, further inquiries can be directed to the corresponding author.
